# Inhaled Antibiotic Therapy in Chronic Respiratory Diseases

**DOI:** 10.3390/ijms18051062

**Published:** 2017-05-16

**Authors:** Diego J. Maselli, Holly Keyt, Marcos I. Restrepo

**Affiliations:** 1 Division of Pulmonary Diseases & Critical Care Medicine, South Texas Veterans Health Care System, San Antonio, TX 78229, USA; masellicacer@uthscsa.edu (D.J.M.); keyt@uthscsa.edu (H.K.); 2 University of Texas Health at San Antonio, San Antonio, TX 78240, USA

**Keywords:** aerosols, cystic fibrosis, bronchiectasis, nontuberculous mycobacteria

## Abstract

The management of patients with chronic respiratory diseases affected by difficult to treat infections has become a challenge in clinical practice. Conditions such as cystic fibrosis (CF) and non-CF bronchiectasis require extensive treatment strategies to deal with multidrug resistant pathogens that include *Pseudomonas aeruginosa*, Methicillin-resistant *Staphylococcus aureus*, *Burkholderia* species and non-tuberculous *Mycobacteria* (NTM). These challenges prompted scientists to deliver antimicrobial agents through the pulmonary system by using inhaled, aerosolized or nebulized antibiotics. Subsequent research advances focused on the development of antibiotic agents able to achieve high tissue concentrations capable of reducing the bacterial load of difficult-to-treat organisms in hosts with chronic respiratory conditions. In this review, we focus on the evidence regarding the use of antibiotic therapies administered through the respiratory system via inhalation, nebulization or aerosolization, specifically in patients with chronic respiratory diseases that include CF, non-CF bronchiectasis and NTM. However, further research is required to address the potential benefits, mechanisms of action and applications of inhaled antibiotics for the management of difficult-to-treat infections in patients with chronic respiratory diseases.

## 1. Introduction

Chronic respiratory diseases that produce bronchiectasis are associated with difficult-to-treat infections that create a real challenge in clinical practice. Difficult-to-treat infections due to multidrug-resistant (MDR) pathogens cause great concern for physicians, caregivers and patients, because these infections are associated with high morbidity, mortality and healthcare system cost. One of the cornerstones of the management of serious and difficult-to-treat infections is the use of antibiotics [1,2,3,4,5,6]. However, the emergence of antimicrobial-resistant pathogens, the lack of newly-developed therapies, and the high cost associated with management of these infections represent major challenges in the care of patients with chronic respiratory diseases.

Patients with chronic respiratory diseases such as cystic fibrosis (CF) and non-CF bronchiectasis may be affected by complex infections by *Pseudomonas aeruginosa*, Methicillin-resistant *Staphylococcus aureus*, *Burkholderia* species and non-tuberculous *Mycobacteria* (NTM) [1,2,3,4,5,6]. Currently, there are limited alternatives available for the management of patients with these serious infections. Inhaled antibiotics have been used to treat respiratory tract infections for several decades [7]. Advantages of inhaled antibiotic administration include the potential to deliver higher drug concentrations at the site of infection without the systemic adverse effects observed with the use of parenteral or oral antibiotic agents. However, clinical and technical issues have limited the advancement of the science in this area of research. Over the past decades there has been increasing interest in development of inhaled antibiotics that may help with the management of (MDR) pathogens, particularly those that are difficult to eradicate or that have a high chance of recurrence. Adjunctive therapies that combine inhaled and systemic antibiotics may potentially increase the efficacy of these medications in the care of patients with MDR pathogens and chronic respiratory disease. This narrative review will describe the currently available evidence regarding the use of inhaled antibiotics for the treatment of difficult-to-treat infections in patients with chronic respiratory diseases that include CF, non-CF bronchiectasis and NTM pulmonary infections.

## 2. Cystic Fibrosis

Cystic fibrosis (CF) is a multisystem autosomal recessive disorder affecting approximately 70,000 people worldwide and 30,000 people in the United States (US) [8]. CF is caused by dysfunction of the cystic fibrosis transmembrane conductance regulator (CFTR) protein, an ion channel located on the apical surface of epithelial cells which is responsible for chloride and bicarbonate transport across the cell membrane [9]. The results of CFTR dysfunction are abnormally thick and viscous secretions in the airways and impaired mucociliary clearance. The natural history of CF is characterized by recurrent lower-respiratory tract infections, often caused by drug-resistant pathogens such as *S. aureus*, *P. aeruginosa*, *Burkholderia cepacia* complex, *Stenotrophomonas maltophilia*, and others [8]. Recurrent infections contribute to a cycle of chronic inflammation, airway destruction and the development of bronchiectasis, leading ultimately to progressive decline in lung function.

However, with advances in diagnostic and therapeutic techniques, patients with CF have an increasing life span [10]. In 2014, there were more adults alive with CF than children for the first time. Inhaled antibiotic therapy has significantly contributed to improved survival [8]. Generally, inhaled antibiotics have been shown to improve lung function, delay decline in lung function, prolong time to exacerbations and improve quality of life in people with CF [10]. Despite these improvements, there is more work to be done: CF continues to cause mortality at an early age; the median predicted survival age in the US in 2015 was 41.6 years [8]. The most common cause of death in patients with CF continues to be respiratory/cardiorespiratory disease, mostly related to infectious complications [8].

One of the most prevalent pathogens in the CF airway is *P. aeruginosa* [8]. More than half of patients in the US with CF have at least one strain of *P. aeruginosa*, including MDR *P. aeruginosa*. However, the prevalence of *P. aeruginosa* has declined over the past decades. Advances in delivery and the increased availability of inhaled antibiotics, as well as the widespread implementation of therapy to eradicate initial acquisition of *P. aeruginosa*, has contributed to this decline [1,11]. As the prevalence of *P. aeruginosa* has decreased, there has been a sharp rise in the prevalence of *S. aureus* in CF airways, including methicillin-resistant *S. aureus* (MRSA). *S. aureus* is now the most prevalent organism in the CF airway. From 2000 to 2015, the prevalence of MRSA increased five-fold with most strains being hospital-acquired infections (approximately two-thirds), compared to community-acquired infections (approximately one-third) [8,12,13,14]. Other common pathogens causing respiratory illness in patients with CF include *Haemophilus influenzae*, which is more common in infants and young patients, *S. maltophilia*, *Achromobacter* species and *B. cepacia* complex [8] (Table 1). More than 10% of patients with CF have cultures positive for mycobacterial species [8].

### 2.1. Pseudomonas aeruginosa and Cystic Fibrosis (CF)

*P. aeruginosa* is the most common pathogen in the airways of patients with CF and is associated with accelerated decline in lung function as well as higher morbidity and mortality [15,16]. Infections due to *P. aeruginosa* range in severity from colonization without an immunologic response to severe pneumonia. However, once acquired, *P. aeruginosa* is difficult to eradicate and patients frequently become chronically infected [1,17]. Over time *P. aeruginosa* strains undergo a phenotypic change to the mucoid phenotype, characterized by production of alginate and associated with even greater difficulty in eradication of the pathogen [1,17]. The Leeds criteria were developed to help researchers describe *P. aeruginosa* infections in patients [18]. By this system, chronic infection is defined as having more than 50% of cultures positive for *P. aeruginosa* in the prior year. In 2015, 30% of patients in the US had chronic *P. aeruginosa* infection [8]. That same year, 17% of patients had intermittent *P. aeruginosa* infection (<50% of cultures positive for *P. aeruginosa*), 29% were free from *P. aeruginosa* in the prior year, and 18% had never had a positive *P. aeruginosa* culture. Chronic infection is associated with increased morbidity and mortality in patients with CF and the natural history of these infections is punctuated by periods of acute pulmonary exacerbations. Currently, the only US FDA-approved indication for inhaled antibiotics is for chronic *P. aeruginosa* infection in patients with CF.

### 2.2. Systemic vs. Inhaled Antibiotics

There are several systemic antipseudomonal agents available for use including extended-spectrum penicillins, aminoglycosides, cephalosporins, fluoroquinolones, monobactams, and others. The most commonly used systemic regimens for treatment of *P. aeruginosa* infections consist of intravenous (IV) β-lactams combined with aminoglycosides [19]. The rationale for combined treatment is based on the knowledge that *P. aeruginosa* strains develop resistance to antimicrobial agents relatively easily [15]. There is a lack of high-quality evidence demonstrating a clear benefit of this approach, nonetheless, it is considered to be standard of care in the US and recommended by the CF Pulmonary Guidelines Treatment of Pulmonary Exacerbations [3].

Systemic delivery of antipseudomonal antibiotics exposes patients to the potential for significant toxicity. For example, tobramycin is an aminoglycoside commonly administered IV for treatment of acute exacerbations of CF. However, IV tobramycin does not penetrate well into the sputum, at a peak reaching only approximately 12% of serum levels. Because bactericidal effect can only be reliably produced with concentrations 25 times the minimum inhibitory concentration (MIC), high doses are required to achieve concentrations inhibitory to *P. aeruginosa* in the airway [20]. These high doses increase the risk of systemic adverse events such as nephrotoxicity and ototoxicity [10,21]. IV administration of broad-spectrum antibiotics also disrupts the normal gut flora, increases risk for secondary infections such as *Clostridium difficile*, and may promote drug resistance [22].

The delivery of antibiotics via inhalation poses significant advantages for treating lower airway infections compared to systemic (oral or IV) therapy. Inhaled therapy allows for targeted delivery of high-concentrations of medications directly to intended the site of activity with minimal systemic absorption and toxicity [23]. In the 1980s, administration of tobramycin via inhalation for the treatment of infections caused by *P. aeruginosa* in CF patients resulted in higher concentrations of the drug at the site of activity with minimal systemic absorption [24,25]. Since then, additional antibiotics have been studied in patients with chronic PA infections, including: tobramycin (available as a solution for inhalation (TSI) or dry-powder inhaler (TIP)), aztreonam for inhalation solution (AZLI), colistin, and inhaled fluoroquinolones. With the increase in prevalence of *S. aureus* in the CF airway, there is a burgeoning increase in development of nebulized vancomycin as well.

These aerosolized antibiotics reduce the frequency of exacerbations, reduce airway bacterial density, improve pulmonary function, and improve quality of life in patients with CF [26]. Therefore, these medications are considered to be standard of care as part of chronic management of pulmonary disease associated with CF, and are recommended by the American Thoracic Society (ATS) in their CF Pulmonary Guidelines [2].

### 2.3. Tobramycin

Tobramycin, an aminoglycoside with activity against *P. aeruginosa*, was one of the first inhaled antibiotics studied in CF (Table 2). Systemic absorption of inhaled tobramycin is low and up to 95% of patients achieve a sputum concentration of drug at least 25 times the MIC, with a median serum/sputum concentration of 0.01 [27]. Such high concentrations are required for effective killing of *P. aeruginosa* because aminoglycosides bind to mucins in sputum and reduce the availability of effective antibiotic [27]. Tobramycin solution for inhalation (TSI) can be administered using the PARI LC PLUS^®^ jet nebulizer and the DeVilbiss Pulmo-Aide^®^ compressor in approximately 20 min or using the PARI eFlow^®^ electronic nebulizer in approximately 7 min [28]. The drug is administered twice daily. It has been well-established that cyclic treatment with TSI is associated with improved pulmonary function, decreased sputum density, fewer hospitalizations and decreased need for systemic antibiotics.

A specific formulation of tobramycin for inhalation was developed in the 1980s and in landmark trials published in the early 1990s, patients with moderate-to-severe lung disease who were treated with TSI had significant improvements in forced expiratory volume in one second (FEV_1_), reduced rates of hospitalization, and decreased hospitalization days compared to patients who received placebo [26,29,30]. Follow-up studies in younger patients and those with milder lung disease demonstrated that treatment with TSI twice daily for 28 days was safe and resulted in decreased density of *P. aeruginosa* in the lower airways, decreased rates of pulmonary exacerbations requiring hospitalization, but without significant impact on lung function [31,32].

The initial TSI trials established the practice of the intermittent 28-day “on/off” regimens for inhaled antibiotics. This design was based on the observation that there was minimal additional improvement in lung function after four weeks of therapy in conjunction with the concern for selection of resistant bacteria. However, in an early phase trial, patients treated with continuous tobramycin had improvement in lung function that remained above baseline for 12 weeks [33]. More recently, the use of continuous therapy either with a single agent or as alternating therapy, has increased in an effort to prevent exacerbations and decline in lung function [34]. A 28-week, multicenter, randomized, double-blind, placebo-controlled trial was conducted to evaluate the use of continuous alternating therapy with TSI and aztreonam compared to intermittent TSI alone [35]. The trial faced difficulty with enrolling patients and did not achieve statistical significance but suggested a potential benefit in reduction of pulmonary exacerbations (by 25%), rates of hospitalization (by 35%), treatment with non-study antibiotics and median time to first exacerbation (175 days vs. 140 days). Further studies are needed to determine the optimal duration of therapy and whether there is a benefit with continuous therapy.

The optimal regimen and duration of treatment for a first positive culture for *P. aeruginosa* is also unclear. The ELITE trial, a multicenter, open-label, randomized study was designed to answer this question in patients with newly-acquired *P. aeruginosa* infection [36]. Patients were randomized to 28 or 56 days of treatment with standard doses of TSI administered twice daily. More than 90% of patients had negative cultures for *P. aeruginosa* one month after the end of treatment without significant difference between the two groups. Patients also remained *P. aeruginosa*-free for more than two years after treatment with no significant difference in the median time to recurrence between the two groups [36].

The role of TSI in acute pulmonary exacerbations is unclear. Small retrospective studies have demonstrated no significant difference between treatment with inhaled or IV antipseudomonal antibiotics during acute exacerbations [37,38,39]. A small pilot study of 20 patients with CF patients chronically infected with *P. aeruginosa* compared 14 days of IV tobramycin vs. TSI 300 mg twice a day [40]. Although the study was small, there was a significant improvement in the time to next exacerbation requiring hospitalization in the TSI group (8.9, mean = 4.7 vs. 4.3, mean = 1.3 months; *p* < 0.001). Patients that received IV therapy developed higher levels of proteinuria and other markers of tubular injury compared to inhaled therapy. Of note, patients in the study were treated with twice- or thrice-daily dosing of IV tobramycin whereas once daily, extended-interval dosing is recommended by CF consensus guidelines based on specific evidence for decreased nephrotoxicity in children. Larger randomized controlled trials are lacking and therefore, current pulmonary guidelines recommend against this treatment approach.

TSI is generally well tolerated; the most common side effects include cough (41–88%), voice alteration (12–16%), and tinnitus (3%) [26,31,32,41]. Symptoms are typically transient and resolve with discontinuation of the medication. However, complaints of tinnitus may be one of the sentinel symptoms of cochlear toxicity and should be carefully evaluated.

Recently, tobramycin inhalation powder (TIP) has been evaluated as an alternative to TSI. The dry powder formulation uses a T-326 inhaler, has shorter administration time and decreases the potential for contamination of the device but with increased side effects and tolerability issues [28]. This type of formulation may be preferred by patients because of the shorter administration duration compared to TSI [42]. With respect to pharmacokinetic properties, similar medication levels were observed with 112 mg of tobramycin powder every 12 h compared to 300 mg of TSI [43]. Konstan et al. reported that TIP formulation was non-inferior to TSI in an open-label study of 553 patients with CF who were over the age of six [44]. Compared to TSI, TIP had similar efficacy in terms of lung function and sputum *P. aeruginosa* density but could be delivered much faster (5.6 min vs. 19.7 min; *p* < 0.001). The rates of cough and overall discontinuation of study drug were much higher in the TIP group, however, despite this, overall satisfaction and quality of life scores were higher in the group of patients who were able to tolerate and were subsequently assigned to the dry-powder formulation [44]. A study evaluated the safety of TIP after one year of exposure (7 cycles) in 62 patients with CF [45]. This presentation of tobramycin was overall well tolerated with no apparent serious adverse events. The most common side effects were cough (15%), impaired hearing (10%) and respiratory tract infections (10%). This safety profile is consistent with prior studies [44,46].

### 2.4. Aztreonam

Aztreonam is a monobactam antibiotic delivered intravenously or via inhalation, with activity against *P. aeruginosa* and other Gram-negative pathogens. Early randomized, double-blind, placebo-controlled trials conducted in patients with CF were short-term but demonstrated positive results with the use of aztreonam solution for inhalation compared to placebo (Table 3). McCoy et al. demonstrated in 211 patients with CF receiving intermittent inhaled tobramycin that administration of inhaled aztreonam twice or three times daily using the PARI eFlow (Altera)^®^ electronic nebulizer for 28 days significantly increased time to next respiratory exacerbation compared to placebo (92 days vs. 71 days; *p* = 0.002), improved FEV_1_ by 6.3–10.3%, and improved quality of life scores compared to placebo [47]. Retsch-Bogart et al. also demonstrated improvement in FEV_1_ and quality of life scores, as well as a decrease in hospital days in patients using inhaled aztreonam compared to placebo (0.5 days vs. 1.5 days; *p* = 0.049) [48].

Given that the use of inhaled antipseudomonal antibiotics for patients with chronic *P. aeruginosa* infection is now well established, long-term, placebo-controlled trials of specific inhaled antibiotics are not feasible. However, a longer, 18-month, open label study in 195 patients with a mean age of 26 years, suggested that long-term use of inhaled aztreonam for 28 days every other month is safe and effective [49]. This study demonstrated improvement in pulmonary function and quality of life scores without an increase in resistance to aztreonam. The findings were more significant with three times a day use compared to twice a day [50]. In addition, a study of 273 individuals with CF aged six years or older demonstrated improved lung function and fewer exacerbations over three 28-day cycles of inhaled aztreonam compared with inhaled tobramycin [51]. These beneficial effects have not been observed in CF patients with other pathogens. For instance, a double-blind, randomized trial evaluated the effects of inhaled aztreonam three times daily for 24 weeks or placebo in 100 CF patients with chronic *B. cepacia* infection [52]. No significant differences were observed in lung function, exacerbation rates, use of antibiotics or hospitalizations. Until further studies are carried out, aztreonam is only recommended for CF patients with *P. aeruginosa* infection.

Inhaled aztreonam is generally well tolerated. The most commonly reported adverse reactions include cough (32–35%), headache (6–11%), bronchospasm (6–10%), nasal congestion (7–10%), nasal congestion (7–10%), and rhinorrhea (7%) [47,48,53]. There are reports of patients having bronchoconstriction with use and, therefore, providers should consider a monitored trial dose particularly in patients with severe lung disease.

### 2.5. Colistin

Colistin belongs to the polymyxin group of antibiotics. It was first discovered in the mid-20th century as a fermentation product of the bacteria *Bacillus colistinus* and acts as a deterrent to interfere with the structure and function of bacterial cell walls [54]. Colistin is bactericidal and active against Gram negative bacteria including *P. aeruginosa*. Nebulized colistin is a preferred inhaled therapy for patients with CF and chronic *P. aeruginosa* in the United Kingdom and has been used for decades in Europe (Table 4). A prospective, double-blind, placebo-controlled study of 40 patients with CF, aged 7–35 years, compared three months of nebulized colistin to placebo and found that patients treated with colistin had improved symptom scores, slower decline in lung function and reduced inflammatory parameters [55]. Hodson et al. compared TSI and colistin in 115 patients with CF who were chronically infected with *P. aeruginosa*. Patients were randomized to receive TSI or colistin twice daily for four weeks [56]. Treatment with TSI resulted in a significant improvement in the primary endpoint of relative change in lung function compared to baseline. There was no significant improvement in the colistin-treated patients but both groups had significant decline in bacterial density [56].

Colistin has also been reformulated as a dry powder inhaler and was found to be non-inferior to TSI in a 24-week, randomized, non-blinded trial of 380 patients with CF [57]. However, both study groups failed to reach the primary endpoint of the trial, which was an improvement in FEV_1_ [57].

The most significant adverse effect reported in association with nebulized colistin is bronchospasm. Bronchoconstriction with a transient decrease in FEV_1_ has been reported in up to 17.7% of patients [56,58,59,60]. This can be mitigated by administration of a short-acting β-2 agonist prior to treatment. Colistin has not been approved for use in the US due to a report of a patient death associated with inhalation of pre-mixed solution of colistin. After mixing colistin with sterile water, it undergoes conversion to the bioactive form which has a component (polymyxin E1) that is toxic to lung tissue. Premixing colistin into aqueous solution and storing it for more than 24 h results in increased concentrations of polymyxin E1 and increased potential for toxicity [61]. Despite this, colistin is used in CF centers in the US for chronic *P. aeruginosa* infection.

### 2.6. Fluoroquinolones

Fluoroquinolones are important antipseudomonal antibiotics and inhaled preparations have been studied in CF patients with chronic *P. aeruginosa* infections (Table 5). Inhaled levofloxacin is the most recently introduced and is currently approved for use in Europe for patients with CF and chronic *P. aeruginosa* infection. Early trials compared varying doses of inhaled levofloxacin (120, 240 mg daily, 240 mg twice daily) to placebo in 151 patients and demonstrated a dose-dependent improvement in lung function and significant decrease in the incidence of acute pulmonary exacerbations over 28 days [62]. More recently, a phase 3, open-label randomized trial compared the safety and efficacy of levofloxacin 240 mg inhaled twice daily to standard doses of TSI in 282 patients with CF who were at least 12 years of age. Between the two groups, there was no difference in lung function or adverse effects at 28 days [63]. Flume et al. compared the safety and efficacy of a 28-day course of levofloxacin inhalation solution to placebo in a multinational, randomized, double-blind trial in 330 patients with CF. Although there was an improvement in the relative change in FEV_1_ percent predicted from baseline (mean difference 1.31%, *p* = 0.01), there was in the patients randomized to levofloxacin compared to the placebo arm [64]. Possible explanations for these results include insufficient antibiotic concentrations, dissimilarities in the study populations or an inappropriate definition of an exacerbation. The most commonly reported adverse events with levofloxacin administration include cough, taste disturbances, tiredness or weakness. It is contraindicated in pregnant or breastfeeding patients, those with epilepsy and those with a history of tendon disorders related to the use of fluoroquinolone antibiotics.

Ciprofloxacin has been formulated into a dry powder for inhalation (DPI) [65,66] and a liposomal form [21,67], and because of its adequate tolerability profile and minimal systemic exposure it has been considered for the treatment of patients with chronic pulmonary conditions and *P. aeruginosa* infection. The DPI form of ciprofloxacin is delivered via a T-326 inhaler and employs the PulmoShere^®^ technology, which uses an emulsion-based spray-drying process to produce highly dispersible low density particles [68]. Ciprofloxacin DPI has been tested in patients with CF with various results [69,70,71,72]. A phase 1, randomized, single-blind, placebo-controlled trial explored the effects of ciprofloxacin DPI in 25 patients with CF and chronic infection with *P. aeruginosa* [69]. Ciprofloxacin was detected at microbiological active concentrations (sputum) and no serious adverse events or changes in pulmonary function were reported. These results led to a phase 2b, randomized, double-blind, controlled trial that studied the effects ciprofloxacin DPI at 32.5 or 48.75 mg twice a day for 28 days or placebo in CF patients colonized with *P. aeruginosa* [71]. Patients treated with ciprofloxacin DPI did not achieve a significant improvement with either dose in FEV_1_ from baseline compared to placebo (*p* = 0.154). The density of *P. aeruginosa* was lower after therapy in the treated group compared to placebo; the mean *P. aeruginosa* colony count expressed as log_10_ CFU/g was 6.73 vs. 7.08 for the 32.5 mg dose (*p* < 0.001) and 6.77 vs. 7.37 for the 48.75 mg dose (*p* = 0.002), but the effects were not sustained after four weeks of therapy. Additional phase 3 studies, with longer treatment duration are required before the role of inhaled ciprofloxacin in CF can be determined. The most common side effects reported with ciprofloxacin DPI are bitter taste (14–94%), bronchospasm (50–67%), headache (17–33%) and cough (3–17%) [69,70,71].

## 3. Non-Cystic Fibrosis Bronchiectasis

Bronchiectasis is a permanent dilation of the airways often associated with chronic respiratory symptoms such as persistent cough, excessive sputum production and recurrent pulmonary infections [4]. In patients without cystic fibrosis (CF), the presence of bronchiectasis can be secondary to a wide range of conditions. Non-CF bronchiectasis (NCFB) has been linked to autoimmune diseases (i.e., rheumatoid arthritis), impaired secretion clearance (i.e., primary ciliary dyskinesia), immunodeficiency (i.e., common variable immunodeficiency), previous infections (i.e., *Mycobacterium tuberculosis*), preexisting pulmonary conditions (i.e., chronic obstructive pulmonary disease) and others [72]. While NCFB was previously considered a rare disease, in the past decades its prevalence has increased [73]. This is likely due to increased physician recognition and the widespread availability of computed tomography, but other factors such as antibiotic prescription practices, and environmental and geographical factors may be influencing these observations [74,75]. The prevalence of NCFB ranges from 500 to 1100 per 100,000 persons, and is more common among women and older individuals [63,76,77,78].

The pathophysiology of NCFB has been well characterized. In the susceptible host, an initial pulmonary insult (i.e., infection or inflammation) will result in a permanent dilation of the airways. These changes in the architecture the airways will affect normal airway secretion clearance. The accumulation of mucus will in turn favor the development of infections, which lead to further inflammation and distortion of the airways. This concept is known as the “vicious cycle” of bronchiectasis [72]. Therefore, pulmonary infections play a pivotal role in the development and worsening of NCBF and their management plays an integral part in the care of these patients. The most common pathogens identified in patients with NCFB are *H. influenzae* (55–29%) and *P. aeruginosa* (28–12%), although in up to a fifth of cases a pathogen cannot be identified [79,80,81,82,83]. Other respiratory pathogens identified in patients with NCFB using cultures, quantitative polymerase chain reaction (PCR) technique, or by ribosomal gene pyrosequencing include *S. maltophilia*, *B. cepacia* complex, *M. catarrhalis*, *S. aureus*, and various species of *Prevotella* and *Veillonella* [81,82,83]. Most recently, the United States Bronchiectasis Research Registry (*n* = 1826) reported that 63% of the patients included in the cohort had evidence of non-tuberculous mycobacteria (NTM) [84]. The most common NTM isolated was *M. avium* complex, followed by *M abscessus/chelonae*. The patients that participated in this study originated from tertiary referral centers with an interest in NTM, which may explain the difference in the reported prevalence of NTM compared to studies from other parts of the world [84].

The type of organism identified in patients with NCFB has important implications. Patients with positive cultures for *P. aeruginosa* have worse outcomes compared to those with other pathogens [85,86,87,88]. For instance, a retrospective study exploring the clinical characteristics and outcomes of 539 patients with NCFB showed that those with positive sputum cultures with *P. aeruginosa* had worse pulmonary function compared to those with other pathogens [85]. Similar findings were reported in a prospective study of 142 patients, in which FEV_1_ and diffusion of carbon monoxide were significantly lower in those with positive testing for *P. aeruginosa* [86]. A Spanish, single-center study of 76 patients with NCFB revealed that accelerated lung function decline was independently associated with chronic colonization with *P. aeruginosa* (odds ratio 30.4, 95% confidence interval 3.8 to 39.4, *p* = 0.005) [87]. Exploring the rates of health care utilization, a longitudinal retrospective observational cohort study of 155 patients with NCFB showed that hospital admissions were significantly higher in a *P. aeruginosa* infected group compared to patients infected with *H. influenzae* (1.3 vs. 0.7 admissions per year, *p* = 0.035) [88]. Moreover, the presence of *P. aeruginosa* has been linked to worse quality of life and greater risk for hospitalization [89,90]. There is also a strong association between increased mortality and the identification of *P. aeruginosa* in patients with NCFB [90,91]. Using a phenotypic cluster analysis based on a microbiologic analysis and the sputum production characteristics, a study showed that NCFB patients with chronic infection with *P. aeruginosa* had significantly more exacerbations, worse pulmonary function and quality of life, and increased markers of inflammation [92]. The reasons why *P. aeruginosa* has such a profound effect on patients with NCFB is multifactorial and related to its virulence factors. This pathogen can survive in wide fluctuations of pH, nutrient limitations and resists multiple classes of antibiotics [93,94]. A key feature of this organism is the ability to form a biofilm, which limits its exposure to host defense mechanisms and antibiotics [93,94]. For these reasons, patients with NCFB require cultures during routine visits as surveillance in addition to periods of exacerbation, as these not only may guide therapy, but may also identify patients at risk for poor outcomes [4].

Antimicrobial therapies continue to play a central role in the therapy of NCFB as maintenance and during exacerbations. Systemic antibiotics may have a higher toxicity profile, require intravenous access, and medical personnel are often employed for appropriate and safe delivery. Inhaled antibiotics are an attractive alternative for patients with pulmonary infections. The objective of inhaled antibiotics is to provide the highest concentrations of active drug at the site of infection without risking systemic toxicity. Various inhaled antibiotics have been studied in patients with NCBF with variable results and while both inhaled ciprofloxacin products are completing phase 3 trials, no inhaled antibiotics have been approved.

### 3.1. Tobramycin

The efficacy of tobramycin in patients with CF and *P. aeruginosa* infections, has led to various studies in NCFB cohorts (Table 6). An initial placebo-controlled, multicenter, double-blind, randomized study explored the efficacy and safety of tobramycin solution in 74 patients with NCFB and *P. aeruginosa* [95]. The patients in the treatment group (*n* = 37) had a significant decreased in the mean *P. aeruginosa* colony forming units (CFU) per gram of sputum compared to no change in the control group (*n* = 37). Additionally, *P. aeruginosa* was eradicated in 35% of patients in the treatment group with substantial improvement in clinical symptoms. No significant differences were observed in the development of tobramycin-resistant strains or in the pulmonary function test results. Patients that received inhaled tobramycin experienced more dyspnea (32% vs. 8%), cough (41% vs. 24%), wheezing (16% vs. 0%) and chest discomfort (19% vs. 0%) compared to placebo, but it did not limit the delivery of therapy. A subsequent study that explored the effects of three cycles of 14 days of tobramycin solution and 14 days off therapy in 41 patients with NCBF and a history of *P. aeruginosa* revealed improvements in the mean pulmonary total symptom severity scores (reduction of 1.5 units, *p* = 0.006) and St. George Respiratory Questionnaire scores (reduction of 9.8 units, *p* < 0.001) [96]. Notably, 22% of the patients withdrew from the study due to adverse events, which more commonly were respiratory: cough (43.9%), dyspnea (34.1%), and increased sputum production (29.3%). A double-blind, placebo-controlled study explored clinical outcomes in 30 patients with NCFB and chronic infection with *P aeruginosa* treated with 300 mg of aerosolized tobramycin or placebo twice daily in two cycles, each for six months, with a one-month washout period [97]. Despite the small sample size of the study, the investigators reported an improvement in the number of admissions (0.15 ± 0.37 vs. 0.75 ± 1.16, *p* = 0.038) and days of admission to the hospital (2.05 ± 5.03 vs. 12.65 ± 21.8, *p* = 0.047) compared to placebo, but no difference was observed in antibiotic use, number of exacerbations, pulmonary function or quality of life markers. Bronchospasm was the most frequently reported adverse event (13%), but responded well to bronchodilator therapy. In addition, inhaled tobramycin has been studied in addition to systemic antibiotics during an acute exacerbation of NCFB. A double-blind, multicenter study evaluated the effects of inhaled tobramycin solution or placebo in addition to oral ciprofloxacin in 53 NCFB patients and known *P. aeruginosa* infection during an acute exacerbation [98]. Patients who received double therapy had a trend towards greater rates of eradication (37.5% vs. 20%, *p* = 0.18) compared to single oral ciprofloxacin, but cure rates and relapse rates were similar in both groups. No systemic adverse reactions were observed, but respiratory adverse events (wheezing) were again reported more frequently in patients that received inhaled tobramycin compared to placebo (50% vs. 15%, *p* < 0.01). Based on the current evidence, the use of inhaled tobramycin cannot be recommended for patients with NCFB on a routine basis or during an exacerbation. Despite improvements in some clinical and microbiologic parameters, the studies have small sample sizes and with relatively short-lived exposures and it remains undetermined if prolonged courses of therapy may induce microbiologic resistance. Respiratory adverse events can be present in up to 50% of the patients and this may limit the use of this therapy, particularly in those patients with reactive airways diseases, such as asthma. Future studies, with larger sample sizes and more prolonged exposures that resemble clinical practice may identify subgroups of patients that have greater benefits while limiting adverse events.

Preliminary data suggest that tobramycin inhalation powder is well tolerated in NCFB, with cough being the most frequently encountered side effect (13%), but larger studies are still needed to evaluate the efficacy and optimal dose of this medication [99].

### 3.2. Ciprofloxacin

Inhaled ciprofloxacin, both DPI [100] and liposomal form [101,102,103,104], has been studied in NCFB (Table 7). A multicenter, phase 2 study of 124 patients with NCFB and positive respiratory cultures for pre-defined potential pathogens received 28 days of DPI ciprofloxacin 32.5 mg twice a day against placebo [100]. Subjects in the treatment group (*n* = 60) had significantly increased rate of pathogen eradication (35% vs. 8%, *p* = 0.001) and an important reduction in total sputum bacterial load counts compared to placebo (*n* = 64). Importantly, a reduction CFUs was observed in subjects with *P. aeruginosa* and *H. influenzae*, the most common pathogens affecting NCFB patients. Adverse events were comparable in both groups, including bronchospasm, which occurred rarely (5.0% vs. 4.7%, *p* = 1.0).

The liposomal form of inhaled ciprofloxacin has potential advantages, including controlled and prolonged release of the drug at the site of action, protection against drug degradation, reduced systemic exposure and augmented cellular uptake [104]. This form of ciprofloxacin is delivered using a PARI LC Sprint^®^ nebulizer and PARI TurboBoy-S^®^ compressor [21,67]. A phase 2 study of 36 patients with NCFB explored the effects of once a day inhaled liposomal ciprofloxacin at two different doses for 28 days [105]. Both doses (150 and 300 mg) were effective at reducing *P. aeruginosa* CFUs in the sputum compared to baseline measures by 3.5 log (*p* < 0.001) and 4.0 log (*p* < 0.001) units, respectively. Patients tolerated the study drug well [101]. Similar findings were observed in a multicenter, randomized, double-blind, placebo-controlled trial (ORBIT-1) of inhaled liposomal ciprofloxacin (100 or 150 mg) for 28 days in 96 NCFB patients [102]. Patients of both treatment arms exhibited significant decrease in *P. aeruginosa* CFUs compared to placebo and the medication was well tolerated. These results lead to the development of a phase 2, multicenter, randomized study (ORBIT-2) testing the effects of inhaled liposomal (150 mg) and free (60 mg) ciprofloxacin or placebo on 42 NCFB patients with more than two bronchiectasis exacerbations in the previous year and positive cultures for *P. aeruginosa* at the time of screening [103]. Compared to placebo (*n* = 22), treatment with inhaled liposomal ciprofloxacin (*n* = 20) delayed time to first pulmonary exacerbation (median 134 vs. 58 days, *p* = 0.057), significantly decreased the sputum bacterial density of *P. aeruginosa* (−4.2 ± 3.7 vs. −0.08 ± 3.8 log_10_ CFU/g, *p* = 0.02), and had a similar adverse effect profile. Two identical trials, ORBIT-3 and ORBIT-4, that included 582 NCFB patients with chronic infection of *P. aeruginosa*, compared the effects of placebo and 48 weeks of the combination of inhaled liposomal (150 mg) and free ciprofloxacin (60 mg) in a 28 days on and 28 days off regimen for six cycles [99]. Patients treated with the liposomal ciprofloxacin had an increase in the median time to first exacerbation that required antibiotics and a decrease in the annual rate of exacerbations (regardless of the need of antibiotics) compared to placebo [99]. As with previous trials, inhaled liposomal ciprofloxacin was associated with reduction in the bacterial load. No improvements in lung function were observed and the treatment was well tolerated with similar rates of adverse events in both study groups [104]. Taking into consideration the findings of the recent trails, inhaled ciprofloxacin has been associated with reductions in the bacterial load and improvement in important clinical outcomes. Hence, inhaled liposomal ciprofloxacin is an attractive treatment option for NCFB for with chronic infection of *P. aeruginosa*.

### 3.3. Other Antibiotics

There is a paucity of studies evaluating inhaled antibiotics other than tobramycin and ciprofloxacin in NCFB. A randomized study evaluated the use of nebulized gentamycin (80 mg) twice daily for 12 months compared to placebo in 65 patients with NCFB [106]. Important inclusion criteria were: positive pathogenic bacteria isolated from sputum cultures, at least two exacerbations the prior year, ability to tolerate nebulized gentamicin, and no chronic use of antibiotics [106]. Inhaled gentamycin was studied using gentamicin injectable solution reconstituted for nebulization using 0.9% saline and the Porta-Neb Ventstream^®^ nebulizer. At the end of the study, the treatment group had higher rates of eradication of *P. aeruginosa* and other respiratory pathogens compared to placebo (30.8% vs. 8.7% and 92.8% vs. 38.5%, *p* < 0.001). Additionally, patients treated with gentamycin experienced fewer exacerbations, had an increased time to first exacerbation, and had improvements in symptom scores. Despite these improvements, none of the treatment effects were sustained after three-month treatment-free follow-up period [106].

The use of aztreonam for inhalation solution has been shown to be effective in patients with CF [47,48]. Because of these observations, two identical randomized multicenter phase 3 trials (AIR-BX1 and AIR-BX2) were designed to evaluate the effects of inhaled aztreonam for four weeks or placebo in patients with NCFB [107]. For this study the Altera^®^ Nebulizer System was used to deliver the study drug. All of the 540 patients included in the trials had positive sputum for susceptible Gram-negative pathogens. Although patients exposed to aztreonam had decrease in sputum bacterial density, this did not translate into significant improvements in respiratory symptoms or time to exacerbation. Adverse events were more frequently reported in the aztreonam groups compared to placebo: dyspnea (46% vs. 35%, *p* < 0.01) and fatigue (30% vs. 20%, *p* < 0.01). The discrepancies between the aztreonam CF studies and the results from the AIR-BX1 and AIR-BX2 trials may be due to suboptimal dosing schemes, different airway clearance regimens, and potential overlap with other diseases in older NCFB patients.

Inhaled colistin has gained interest for the treatment of NCFB because of its antipseudomonal properties. A study explored the effects of inhaled colistin (1 million IU twice a day for six months) vs. placebo in 144 NCFB patients and chronic infection with *P. aeruginosa* [108]. Colistin was studied using the I-neb^®^ adaptive aerosol delivery device, which monitors the time and peak flow of the initial three breaths during the nebulization and then delivers therapy intermittently at the start of inspiration to optimize drug delivery [109]. Using this device, therapy is completed in approximately three minutes. The use of inhaled colistin failed to meet the primary endpoint of time to exacerbation versus placebo (165 days vs. 111 days, *p* = 0.11) [108]. In a post-hoc analysis, in those patients considered adherent to therapy, inhaled colistin reduced the median time to exacerbation (168 days vs. 103 days, *p* = 0.038) and improved respiratory symptoms. Adverse events were similar in both study arms (total adverse events: 64% in the colistin group vs. 54% in the placebo group, *p* = 0.25) [108]. In another study, colistin (1 million IU twice a day for 12 months) was compared to conventional therapy in 39 patients with NCFB and chronic infection with *P. aeruginosa* [110]. Patients that received therapy did not experience any improvement in exacerbation rates. Notably, 25% of the patients of the treatment group stopped the therapy due to adverse effects, mainly respiratory. The discrepancies of tolerability between these two studies may be related to the age of the patients included (mean age 59.2 years vs. 77.7 years) and the duration of the study (6 months vs. 12 months) [108,110]. Future, longer duration studies with an emphasis on dose ranging are still needed to understand the potential benefits of inhaled gentamycin, aztreonam and colistin in NCFB. Based on the current evidence these therapies cannot be recommend for routine use in patient with NCFB.

## 4. Non-Tuberculous Mycobacteria

Nontuberculous mycobacteria (NTM) are common pathogens found in the environment and isolated from many parts of the world [111]. Over past decades increasing rates of human disease secondary to NTMs have been reported [112,113,114]. NTMs could affect multiple organs, but pulmonary disease is the most common reason for clinical symptoms as a result of NTM inhalation. However, not always the identification of NTMs determine pulmonary disease. Several scientific organizations have published evidence based guidelines for the diagnosis and management of NTM disease [5,6]. The distribution of NTM species varies according to the geographic reasons, but most of the published data are reported from Europe (United Kingdom), North America (United States of America and Canada), Israel and Japan. The most commonly identified NTM that causes lung disease are *Mycobacterium avium* complex (MAC), *M. abscessus* complex (MABSC) and *M. kansasii*. In the US, inhaled antibiotics are used in up to 10% of the patients with NTM infections [84].

NTM infection are commonly found in patients with risk factors such as chronic lung diseases such as COPD, asthma, alpha-1 antitrypsin deficiency, CF, non-CFB, primary ciliary dyskinesia, and allergic bronchopulmonary aspergillosis [112]. However, patients without pre-existing lung disease such as the classic report of “Lady Windermere Syndrome”, that occurs in white, tall, thin women with pectum excavatum and mitral valve prolapse has been described. These patients usually present with a middle lobe syndrome with bronchiectasis. In addition, NTM lung disease has been found in conditions such as GERD, immunodeficiency and the use of some medications (e.g., immunosuppressive medications, proton pump inhibitors) [84]. Several studies suggest that patients with CF bronchiectasis managed with inhaled antibiotics may be prone to NTM infection. The exact mechanism is unclear, but microbiome balance may play a role, and changes induced by inhaled antibiotics may promote the emergence of NTM in patients with CF, that were initially suppressed by other bacterial species that competed for the same lung environment. However, a study that included 30 MABSC cases and 60 NTM negative CF patients found no association between inhaled antibiotics and MABSC infection [115].

Treatment of pulmonary NTM, especially *M. abscessus*, is complex, requiring multiple systemic antibiotics for long periods of time. However, there is important toxicity and limited efficacy adds to the level of complexity in patients who do not respond to initial therapy, and who have refractory disease or recurrence. The data regarding the use of inhaled antibiotics for patients with NTM is limited. Olivier et al. identified 20 patients with bronchiectasis treated with inhaled amikacin for refractory NTM lung disease [116]. The dosing scheme selected used consisted of an initial dose of 250 mg once daily, followed by 250 mg twice daily after two weeks if no dysphonia was reported. Patients were then told to increase the dose to 500 mg twice daily after two weeks if tolerated. The patients had positive cultures for *M. abscessus* (*n* = 15) and MAC (*n* = 5). The patients received a median of duration therapy of 60 months (ranges from 6 to 190 months) and a follow-up of 19 months. Forty percent of patients had a least one culture, but only 25% (5/20 patients) had persistent negative cultures throughout the follow up period. Symptomatology improved, unchanged and worsened in 45%, 35% and 20%, respectively. Radiological worsening on chest CT scans was noted in 55%, with improvement in 30% and no change in 15%, respectively. Adverse events limited the continuation of inhaled amikacin in seven patients (35%), mainly due to ototoxicity (10%), hemoptysis (10%), and less commonly nephrotoxicity, persistent dysphonia and vertigo (one patient for each adverse event). This observational study suggests that in patients who fail standard systemic therapy, the use of inhaled amikacin may bring some benefit, but with an important rate of adverse events, that should be explained to the patients [116]. Some experts, recommend inhaled amikacin as a step-down therapy in patients presenting drug toxicity with systemic antibiotics. The British Thoracic Society Guidelines currently available for public consultation recommend that MABSC lung disease patients should receive an initial phase of systemic antibiotic treatment followed by a continuation phase of a combined regimen of inhaled and/or oral antibiotics. The recommendation was graded D based on evidence level 3 or 4 (e.g., non-analytical studies, case series, case reports and expert opinion). In conclusion, inhaled amikacin should be considered in place of IV amikacin when systemic administration is impractical, contraindicated or long-term treatment with aminoglycosides is required, particularly for patients with pulmonary disease due to MABSC, MAC, *M. xenopi*, and *M. malmoense*, For patients with CF, the CF foundation and the ECFS [6] recommend that the continuation phase with oral macrolide therapy should be combined with inhaled amikacin and in addition 2–3 oral antibiotics such as minocycline, clofazimine, moxifloxacin, and linezolid.

Recent hope has been given to a novel formulation of liposomal amikacin that may prevent the adverse events related to free amikacin. Rose et al. investigated the activity of an inhaled formulation of liposomal amikacin in an in vitro and in vivo murine model of NTM infection [117]. Macrophage monolayers were infected with MAC and MABSC and treated with liposomal amikacin vs. free amikacin for four days assessing bacterial survival. The authors found that liposomal amikacin was more effective in eliminating intracellular MAC and MABSC compared to free amikacin [117]. In the in vivo model, inhaled liposomal amikacin showed similar reduction of MAC in the lungs as systemic amikacin and no development of acquired resistance [117]. Olivier KN and collaborators performed a double-blind phase II randomized controlled trial that assessed the efficacy and safety of once-daily (590 mg) inhaled liposomal amikacin for the treatment of MAC and MABSC lung disease compared to placebo [118]. The modified intent-to-treat analysis of 89 patients with MAC or MABSC showed that despite the primary endpoint of baseline to Day 84 change on a semiquantitative mycobacterial growth scale was not achieved. Improvement of other endpoints such as sputum conversion, six-minute-walk distance and limited systemic toxicity were observed among subjects refractory MAC lung disease treated with inhaled liposomal amikacin versus placebo [118].

Most recently, the applications of liposomal ciprofloxacin in NTM have been investigated in pre-clinical studies. The efficacy of two different formulations inhaled liposomal ciprofloxacin, Dual Release Ciprofloxacin for Inhalation (DRCFI) and Ciprofloxacin for Inhalation (CFI), was compared to a non-liposomal ciprofloxacin solution and empty liposomes (control) in mice infected intranasally with MAC subspecies Hominissuis [119]. One week after infection, the different formulations were administered intranasally daily for three weeks. Treatment DRCFI or CFI resulted in significant reduction in CFUs from (1.1 ± 0.5) × 10^7^ to (2.5 ± 0.6) × 10^6^ and (2.3 ± 0.4) × 10^6^, respectively. Treatment with the solution of non-liposomal ciprofloxacin and empty liposomes resulted in no changes in the bacterial load. A subsequent, in vitro study demonstrated that a CFI was able to inhibit MAC subspecies *Hominissuis* microaggregates and biofilm formation on plastic surfaces and cultured epithelial cells, providing further evidence of the antimicrobial properties of this preparation [120]. Similar reductions in biofilm formation after treatment with liposomal ciprofloxacin were observed in macrophage monolayers infected with *M. avium*, *M. abscessus* and MAC subspecies *hominissuis* [121]. These observations are promising and warrant further in vivo study, particularly given the urgent need for therapeutic alternatives in these difficult to eradicate pathogens.

In conclusion, there are limited data regarding the use of inhaled antibiotics for the management of patients with NTM pulmonary infections. However, as suggested previously, clinicians treating NTM pulmonary infections should consider the use of inhaled antibiotics when systemic administration is impractical or impossible, aminoglycosides are contraindicated due to systemic adverse effects, long term therapies are needed or palliation of symptoms is the goal of treatment.

## 5. Future Directions

The use of inhaled antibiotics has increased over the last decades in respiratory conditions and the scope of their use is still not completely understood in CF, NCFB and NTM pulmonary infections. In CF, there is an increased interest in the applications of inhaled antibiotics to treat Gram-positive infections given the increase rate of these infections [8]. For instance, an ongoing, double-blind, comparator-controlled, randomized study will evaluate the use of nebulized vancomycin vs. placebo in CF patients with chronic infection with MRSA [122]. Another area of interest is the effects of combining different types of antibiotics. Post hoc analyses of a dataset of previous trial of CF patients treated with tobramycin explored the effects of concomitant use oral azithromycin by study subjects on clinical outcomes [123,124]. Azithromycin was shown to potentially reduce the antimicrobial effects of tobramycin possibly by inducing bacterial stress responses [123,124]. These results have important implications as a significant number of patients with CF utilize oral macrolides and inhaled antibiotics. Future studies are still required to elucidate the complex interactions that may occur with the combination of antibiotics.

Although the results of the recent trials regarding the benefit of inhaled antibiotics in NCFB have not led to the routine use of these therapies, there are still areas of uncertainty that deserve further evaluation. NCBF is a heterogeneous disease and further classifying patients beyond the chronic infection (or not) of *P. aeruginosa* might be required to identify better target subgroups [92]. Adverse events may limit the use of some of these antibiotics, particularly in the elderly, so dose ranging studies and better patient selection algorithms are still warranted. There has been success with oral macrolides in the reduction of exacerbation rates patients with NCFB, however it is unclear the effect of these chronic antibiotic administration on the microbiome [125,126]. Future studies may explore the use of inhaled antibiotics in conjunction with oral therapies (possibly at lower doses) to maximize antimicrobial effects while limiting toxicity [98]. It is not clear if the use oral macrolides in NCFB may have a similar impact on the antibacterial properties of inhaled therapies as seen in CF patients. It is imperative to better understand the factors that could explain why some inhaled antibiotics are effective in CF compared to NCFB. Recognition of these disease characteristics may further aid the clinicians in the selection and treatment of appropriate inhalational therapies for these patients with NCFB.

The success in development of liposomal formulations of antibiotics has promoted new avenues of research in the delivery of lung-targeted medications. Their effects on infected macrophages and biofilms have promising applications in CF, NFCF, and pulmonary NTM infections [121]. In addition to lipid nanocarriers, other polymers have shown promise in the controlled delivery of medications via permeabilization through shell hydrolysis and medium dissolution [127,128]. Through these mechanisms it may be possible to better tailor antibiotic release rates improving the duration of the delivery while achieving target peak concentrations [128,129].

Finally, as our understanding of the role of the microbiome in the outcomes of patients with bronchiectasis increases it might be possible to better select antibiotic strategies. There is increasing evidence that bacterial communities interact with one another and the effects of antibiotics in these interactions are incompletely understood. Because patients with CF, NCFB, and NTM infections often receive IV and oral antibiotics in conjunction with inhaled antibiotics, the interactions between the microbial populations are particularly relevant. Newer, more sensitive molecular techniques are able to identify microorganisms that may not be identified with standard methods [82,130]. It is remains unclear how this information may guide the clinician when selecting antibiotic therapy, but might be important in patients in which microbiome studies reveal that *P. aeruginosa* is not the dominant pathogen. Future studies are needed to explore the benefits of inhaled antibiotics in subgroups of patients with bronchiectasis stratified according to microbiome signatures.

## 6. Conclusions

Great advances have been made in the field of inhaled antibiotics, especially in CF and NCFB. As technology continues to improve the delivery of these medications, clinicians will require balancing the potential benefits of inhaled antibiotics with toxicity and the development of resistance. This is of particular importance in patients with CF, which are now living longer because of the availability of treatments targeting specific gene mutations and will require prolonged antibiotic therapy. Future studies are still required to determine which subgroups of patients with NCFB have the highest likelihood of benefits with inhale antibiotics. The applications of inhaled antibiotics in NTM infections are less understood, and further studies are needed to establish the role of these treatments in this patient population. Combination therapy of oral and inhaled antibiotics may lead to important interactions that are only partially understood. The results of ongoing trials evaluating the use of inhaled antibiotics are eagerly awaited to establish if these can be added to the armamentarium of therapies for patients with CF, NCFB and NTM infections.

## Figures and Tables

**Table 1:** Common pathogens in patients with cystic fibrosis (CF) and median age of first infection (CFFPR 2015).

Pathogen	Percent with Infection	Median Age in Years at First Infection
*S. aureus*	70.6	3.6
*P. aeruginosa*	47.5	5.5
methicillin-resistant *S. aureus* (MRSA)	26.0	11.9
*H. influenzae*	15.5	2.6
*S. maltophilia*	13.6	10.0
multi-drug resistant *P. aeruginosa* (MDR-PA)	9.2	22.4
*Achromobacter* sp.	6.1	14.3
*B. cepacia* complex	2.6	19.9

**Table 2:** Studies of inhaled tobramycin in CF patients with *P. aeruginosa* present in sputum.

Study/Year	Preparation	Dose/Frequency	Duration	Patient Population	Key Outcomes after Treatment
MacLusky 1989 [30]	TSI	80 mg/TID	32 months	*n* = 27	Stability in pulmonary function, controls showed decline
Smith 1989 [33]	TSI	600 mg/TID	12 weeks	*n* = 22	Improved symptoms, decrease in bacterial density
Ramsey 1993 [29]	TSI	600 mg/TID	12 weeks, 28 days on, 28 days off	*n* = 71	Improved pulmonary function
Ramsey 1999 [26]	TSI	300 mg/BID	24 weeks (on/off every 28 days)	*n* = 520	Improved pulmonary function and decreased hospitalizations
Gibson 2003 [31]	TSI	300 mg/BID	28 days	*n* = 21	Treatment reduced lower airway *P. aeruginosa* density
Murphy 2004 [32]	TSI	300 mg/BID	28 days on, 28 days off (7 cycles)	*n* = 184, mild lung disease	Decreased hospitalization rates
Konstan 2011 [44]	TIP or TSI	112 mg/BID or 300 mg/BID	28 days on, 28 days off (3 cycles)	*n* = 517	Comparable efficacy, but greater satisfaction with inhalation powder
Galeva 2013 [46]	TIP	112 mg/BID	28 days on, 28 days off (1 cycle)	*n* = 62	Trend towards improvement in the lung function

BID, twice daily; TID, three times daily; TSI, tobramycin solution for inhalation; TIP, tobramycin inhalation powder.

**Table 3:** Studies of inhaled aztreonam in CF patients with *P. aeruginosa* present in sputum (or *Burkholderia* spp.).

Study	Preparation	Dose/Frequency	Duration	Patient Population	Key Outcomes after Treatment
McCoy 2008 [47]	AZLI	75 mg/BID or TID	28 days with 56 days of follow-up	*n* = 211, receiving inhaled tobramycin	Decreased exacerbation rates, improved lung function and respiratory symptoms
Retsch-Bogart 2009 [48]	AZLI	75 mg/TID	28 days	*n* = 164	Improved lung function and quality of life scores, and decreased number of hospital days
Oermann 2010 [49]	AZLI	75 mg/BID or TID	28 days on, 28 days off (up to 9 cycles)	*n* = 195	TID-treated patients demonstrated greater improvements in lung function and respiratory symptoms
Assael 2013 [51]	AZLI or TSI	75 mg/TID (AZLI) or 300 mg/BID (TSI)	28 days on, 28 days off (up to 9 cycles)	*n* = 273	AZLI-treated patients experienced improved lung function compared to TSI
Tullis 2014 [52]	AZLI	75 mg/TID	24 weeks	*n* = 100, *Burkholderia* spp. present in the sputum	No improvement in lung function
Flume (2016) [35]	AZLI + TSI	75 mg/TID + 300 mg/BID	Alternating 28 days of tobramycin, 28 days of aztreonam for 28 weeks	*n* = 90	Trend towards a reduction in exacerbations and hospitalizations

AZLI, aztreonam solution for inhalation; BID, twice a day; TID, three times a day; TSI, tobramycin solution for inhalation.

**Table 4:** Studies of inhaled colistin in CF patients with *P. aeruginosa* present in sputum.

Study	Preparation	Dose/Frequency	Duration	Patient Population	Key Outcomes after Treatment
Jensen 1987 [55]	CSI	1 million units/BID	3 months	*n* = 40	Improvements in symptom scores and slower decline in lung function
Hodson 2002 [56]	CSI or TSI	80 mg/BID(CSI) or 300 mg/BID(TSI)	4 weeks	*n* = 115	TSI improved lung function but CSI did not. Both decreased bacterial load
Schuster 2013 [57]	CDP or TSI	1.6 million units/BID (CDP) or 300 mg/BID (TSI)	28 days on, 28 days off (3 cycles)	*n* = 380	CDP was non-inferior to TSI, but the primary endpoint regarding lung function was not reached

CSI, colistin solution for inhalation; CDP, colistin dry powder; TSI, tobramycin solution for inhalation; BID, twice a day.

**Table 5:** Studies of inhaled fluoroquinolones in CF patients with *P. aeruginosa* present in sputum.

Study	Preparation	Dose/Frequency	Duration	Patient Population	Key Outcomes after Treatment
Geller 2011 [62]	LSI	120 or 240 mg daily or 240 mg BID	28 days	*n* = 151	Dose dependent increase in lung function and decrease in exacerbations
Stuart Elborn 2015 [63]	LSI or TSI	240 mg/BID (CSI) or 300 mg/BID (TSI)	28 days on, 28 days off (3 cycles)	*n* = 282	LSI was non-inferior to TSI with regards to lung function
Flume 2016 [64]	LSI	240 mg/BID	28 days	*n* = 330	Improvement in lung function but no difference in time to next exacerbation
Dorkin 2015 [71]	CiDP	32.5 mg or 48.75 mg/BID	28 days	*n* = 286	No significant improvements in lung function compared to placebo

BID, twice a day; CiDP, ciprofloxacin dry powder; LSI, levofloxacin solution for inhalation; TSI, tobramycin solution for inhalation.

**Table 6:** Studies of inhaled tobramycin in NCFB patients with *P. aeruginosa* present in sputum.

Study/Year	Preparation	Dose/Frequency	Duration	Patient Population	Key Outcomes after Treatment
Barker 2000 [95]	TSI	300 mg/BID	28 days	*n* = 74	Decrease in bacterial load
Scheinberg 2005 [96]	TSI	300 mg/BID	14 days on, 14 days off (3 cycles)	*n* = 41	Improvements in respiratory symptoms
Drobnic 2005 [97]	TSI	300 mg/BID	6 months	*n* = 30	Improvement in the number of admissions and days of admission to the hospital
Bilton 2006 [98]	Oral ciprofloxacin or oral ciprofloxacin + TSI	750 mg/BID (ciprofloxacin) 300 mg/BID (TSI)	2 weeks	*n* = 53, with an acute exacerbation	Double therapy resulted in a trend towards greater rates of eradication, but cure rates and relapse rates were similar in both groups

BID, twice a day; TSI, tobramycin solution for inhalation.

**Table 7:** Studies of inhaled ciprofloxacin in NCFB patients with *P. aeruginosa* present in sputum.

Study/Year	Preparation	Dose/Frequency	Duration	Patient Population	Key Outcomes after Treatment
Wilson 2013 [100]	CiDP	32.5 mg/BID	28 days	*n* = 24	Reduction in the bacterial load
Bilton 2010 [101]	ILC	150 mg or 300 mg/daily	28 days	*n* = 36	Both doses resulted in a reduction in the bacterial load
Bilton 2011 [102]	ILC	100 mg or 150 mg/daily	28 days	*n* = 96	Both doses resulted in a reduction in the bacterial load
Serisier 2013 [103]	ILC + CiSI	150 mg (ILC) + 60 mg (CiSI)/daily	28 days on, 28 days off (3 cycles)	*n* = 42	Delayed time to first pulmonary exacerbation and reduction in the bacterial load
Haworth 2017 [104]	ILC + CiSI	150 mg (ILC) + 60 mg (CiSI)/daily	28 days on, 28 days off (6 cycles)	*n* = 582	Increase in the median time to first exacerbation that required antibiotics and a decrease in the annual rate of exacerbations (regardless of the need of antibiotics)

BID, twice daily; CiDP, ciprofloxacin dry powder; CiSI, ciprofloxacin solution for inhalation; ILC, inhaled liposomal ciprofloxacin.
